# Low-dose dobutamine cardiovascular magnetic resonance segmental strain study of early phase of intramyocardial hemorrhage rats

**DOI:** 10.1186/s12880-021-00709-x

**Published:** 2021-11-20

**Authors:** Rui Xia, Bo He, Tong Zhu, Yu Zhang, Yushu Chen, Lei Wang, Yang Zhou, Jichun Liao, Jie Zheng, Yongmei Li, Fajin Lv, Fabao Gao

**Affiliations:** 1 Department of Radiology, The First Affiliated Hospital of Chongqing Medical University, Yuzhong District, Chongqing, 400016 China; 2 Department of Radiology, West China Hospital, Sichuan University, 37 Guo Xuexiang Road, Wuhou District, Chengdu, 610041 Sichuan China; 3 Department of Radiology, TongJi Hospital, TongJi Medical College, HuaZhong University of Science & Technology, Hankou, 430030 Wuhan China; 4 Department of Pathology, Chongqing Medical University, Yuzhong District, Chongqing, 400016 China; 5 Mallinckrodt Institute of Radiology, Washington University School of Medicine in St. Louis, Saint Louis, MO 63110 USA

**Keywords:** Myocardial infarction, Reperfusion, Magnetic resonance imaging, Dobutamine

## Abstract

**Background:**

This study investigates the segmental myocardial strain of the early phase of intramyocardial hemorrhage (IMH) caused by reperfused myocardial infarction (MI) in rats by low-dose dobutamine (LDD) cardiovascular magnetic resonance (CMR) feature-tracking.

**Methods:**

Nine sham rats and nine rats with 60-min myocardial ischemia followed by 48-h reperfusion were investigated using CMR, including T2*-mapping sequence and fast imaging with steady-state precession (FISP)–cine sequence. Another FISP–cine sequence was acquired after 2 min of dobutamine injection; the MI, IMH, and Non-MI (NMI) areas were identified. The values of peak radial strains (PRS) and peak circumferential strains (PCS) of the MI, IMH and NMI segments were acquired. The efficiency of PRS and PCS (EPRS and EPCS, respectively) were calculated on the basis of the time of every single heartbeat.

**Results:**

The PRS, PCS, EPRS, and EPCS of the sham group increased after LDD injection. However, the PRS, PCS, EPRS, and EPCS of the IMH segment did not increase. Moreover, the PRS and PCS of the MI and NMI segments did not increase, but the EPRS and EPCS of these segments increased. The PRS, PCS, EPRS, and EPCS of the IMH segment were lower than those of the MI and NMI segments before and after LDD injection, but without a significant difference between MI segment and NMI segment before and after LDD injection.

**Conclusions:**

LDD could help assess dysfunctions in segments with IMH, especially using the efficiency of strain. IMH was a crucial factor that decreased segmental movement and reserved function.

## Background

Myocardial revascularization reduces the infarction size and the incidence of long-term adverse events in clinical practice. However, myocardial revascularization itself can also lead to intramyocardial hemorrhage (IMH), which has been recognized as a poor prognostic factor in myocardial infarction (MI) for the adverse effect of left ventricular (LV) remodeling [1]. A limited number of studies have demonstrated segmental dysfunctions in the presence of IMH in patients with reperfused acute myocardial infarction [2–5]. Low dose dobutamine (LDD) stress was well established for the detection of salvaged myocardial recovery after reperfusion [6, 7]. However, it is unknown whether the presence of IMH has significant impact in local myocardial contractility, at rest or during dobutamine stress.

Feature-tracking (FT) cardiac magnetic resonance imaging (MRI) is a post-processing technique that is increasingly being employed to assess global and regional myocardial stain and strain efficiency. Tomas et al. have confirmed the reproducibility of FT for strain analysis in small animals (mice) [8], but no information exists about local strains in the presence of IMH using LDD-CMR in the early phase of reperfused MI rats. Hence, this study assesses FT based LV strain and strain efficiency using LDD-CMR and tests its ability to detect segmental dysfunction in the presence of IMH.

We designed two groups, namely, the reperfused MI group and the sham group, and hypothesized that this MRI strain analysis at rest and with LDD could discriminate IMH, MI, and non-MI (NMI) segments in the reperfusion group and discriminate these segments in the sham group.

## Methods

### Animal models

All experimental procedures were approved by the experimental Animal Ethics Committee of West China Hospital, Sichuan University (Chengdu, China). Nineteen female Sprague–Dawley rats (Chengdu Dossy Experimental Animal Co.,Ltd.) with body weights ranging from 250 to 300 g were divided into two groups: the reperfusion (n = 10) and sham control groups (n = 9). Before surgery, the rats were intraperitoneally anesthetized using sodium pentobarbital, and respiration was maintained using a rodent ventilator. A real-time electrocardiogram (ECG) was monitored throughout surgery.

Thoracotomy was performed to introduce the coronary occlusion. The chest was opened at the fourth intercostal space to expose the heart, the pericardium was opened using forceps, and a 6.0 suture was passed underneath the left anterior descending coronary artery at a location 2 mm proximal to the ostium of the coronary artery. Coronary occlusion was achieved by tightening the suture over a 3.0 suture. The success of occlusion was confirmed by the pale appearance of the myocardial apex area and immediate changes in the ECG profiles, including a significant increase in the amplitude of the QRS complex and ST-segment elevation [9].

In the early reperfusion model, all occlusions were maintained for 60 min, followed by reperfusion [10]. Reperfusion was achieved by untying the knot and releasing the suture from occlusion. The success of reperfusion was confirmed by ECG changes including further ST-segment elevation followed by gradual recovery of the ST-segment. In the sham surgery model, no occlusion after thoracotomy was performed.

### MRI protocols

MRI was performed 48 h after surgery in both groups [10]. All MRI protocols were implemented using a 7.0 T MR system (BRUKER BIOSPEC 70/30). Each rat was anesthetized with isoflurane (2%–3%) in a small container, and anesthesia was maintained using a mixture of 100% oxygen and isoflurane (1%–2%), administered through a small mask during MRI. Body temperature was monitored using a rectal temperature probe and maintained at 37 °C using a heating blanket. Each rat was placed in the prone position within a surface coil. The ECG signal was obtained from three subcutaneous copper needles inserted in the left forelimb, hind limb, and right forelimb. The respiration signal was acquired from a respiratory pillow (SA Instruments Inc.) placed under the rat [9].

**Fig. 1:**
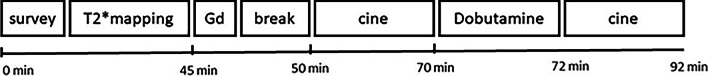
The time points of the examination

Scout imaging was initially performed using a gradient-echo sequence to localize the short-axis images at the middle level of the LV. Multi-slice cine images were obtained to cover the entire heart for functional analysis. After maintaining the respiratory rate at 30–50 cycles per minute by altering the isoflurane concentration, more than five (depending on the size of the heart) single-slice multi-echo T2*-mapping images were acquired on the short-axis slices during the mid-diastolic phase and end-inspiratory period using both ECG and respiratory gating systems. The imaging parameters were as follows: late gadolinium enhancement (LGE) imaging was performed by fast imaging with steady-state precession (FISP–cine on the same slice locations 10 min after the manual injection of gadolinium (Gd)–diethylenetriamine penta-acetic acid (Magnevist, Bayer Health Care Pharmaceuticals, 0.15 mmol/kg). Then, the same FISP–cine was repeated in identical planes after 2 min dobutamine (Gadovist, Bayer) injection at a rate of 10 mg/kg/min. Heart rate was measured before MR scan; before LDD injection; and 2, 20, and 30 min after LDD injection. Figure 1 shows the course of the examination.

The imaging parameters included: T2*-mapping: FA (Flip angle) = 30°, TR/TE = 1000 ms/3.5,7,10.5,14,17.5,21,24.5,28 ms, Matrix size = 192 × 192, FOV = 50 × 50 mm, and slice thickness = 1.5 mm without slice gap. LGE: TR/TE = 5.2 ms/1.8 ms, FA = 25°, matrix size = 256 × 256, FOV = 50 × 50 mm, slice thickness = 1.5 mm, 25 frames for each slice.

### Histology

Following MRI evaluation, the rats were euthanized using potassium chloride (3 mol/L, 0.5 mL), and the hearts were rapidly excised. Each heart was cut into five or more transverse slices from the apex to the base; these slices measured approximately 1.5 mm in thickness to match the MRI slices. These slices were then incubated in 4% paraformaldehyde for hematoxylin and eosin staining.

### Data analysis

The strain data were analyzed using commercially available software (Circle Cardiovascular Imaging, Inc.). The peak global radial strains (PGRS), peak global circumferential strains (PGCS), peak radial strains (PRS), and peak circumferential strains (PCS) were derived using an FT analysis of the LV short-axis. The efficiency of PRS and PCS (EPRS and EPCS, respectively) were calculated using the following formula: PRS (PCS)/R-R interval. The analysis was based on the six segmentation created consisting of six hexahedral elements corresponding to the anteroseptal, anterior, anterolateral, posterolateral, posterior, and poster septal walls.

**Fig. 2:**
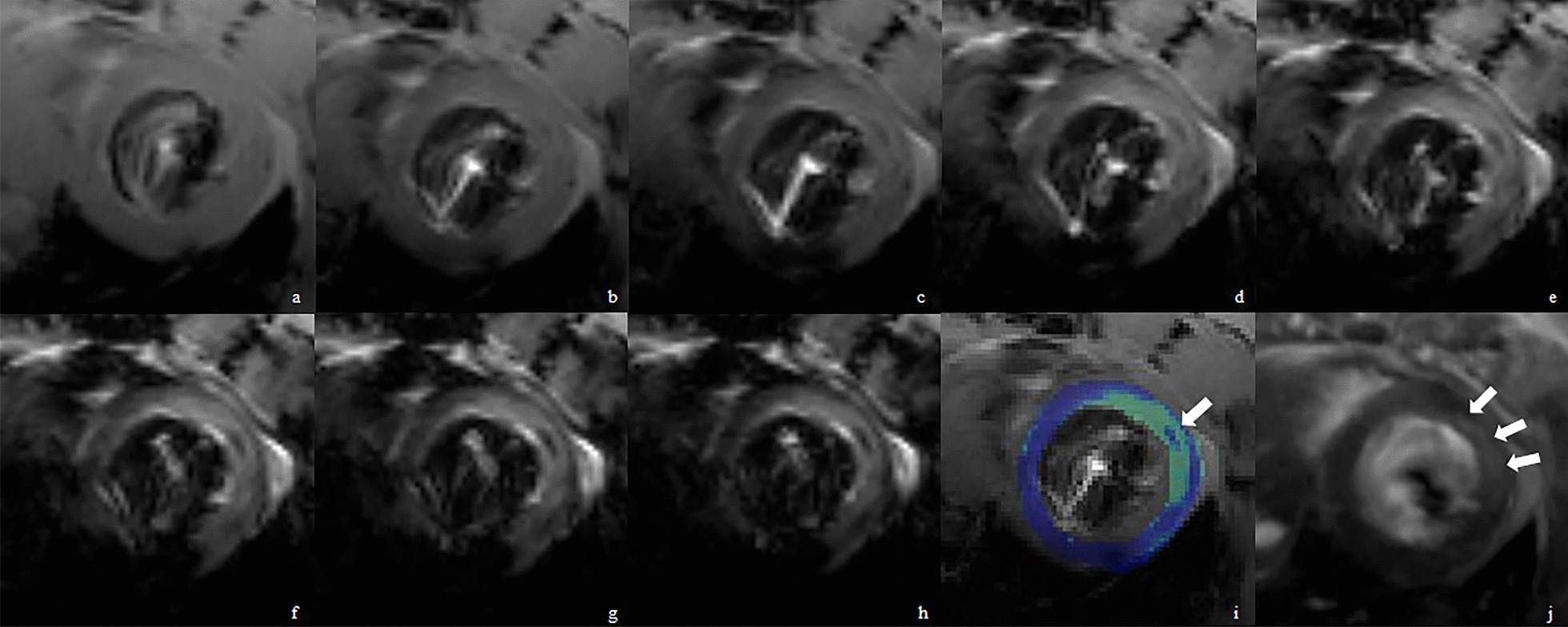
IMH area showed by T2*-weighted images (**a**–**h**, TE = 3.5, 7, 10.5, 14, 17.5, 21, 24.5, 28 ms) and color coded area (**i**, arrow). The myocardial infarction area (arrows) is shown in a LGE sequence (**j**). *IMH* intramyocardial hemorrhage, *MI* myocardial infarction, *LGE* late gadolinium enhancement

**Fig. 3:**
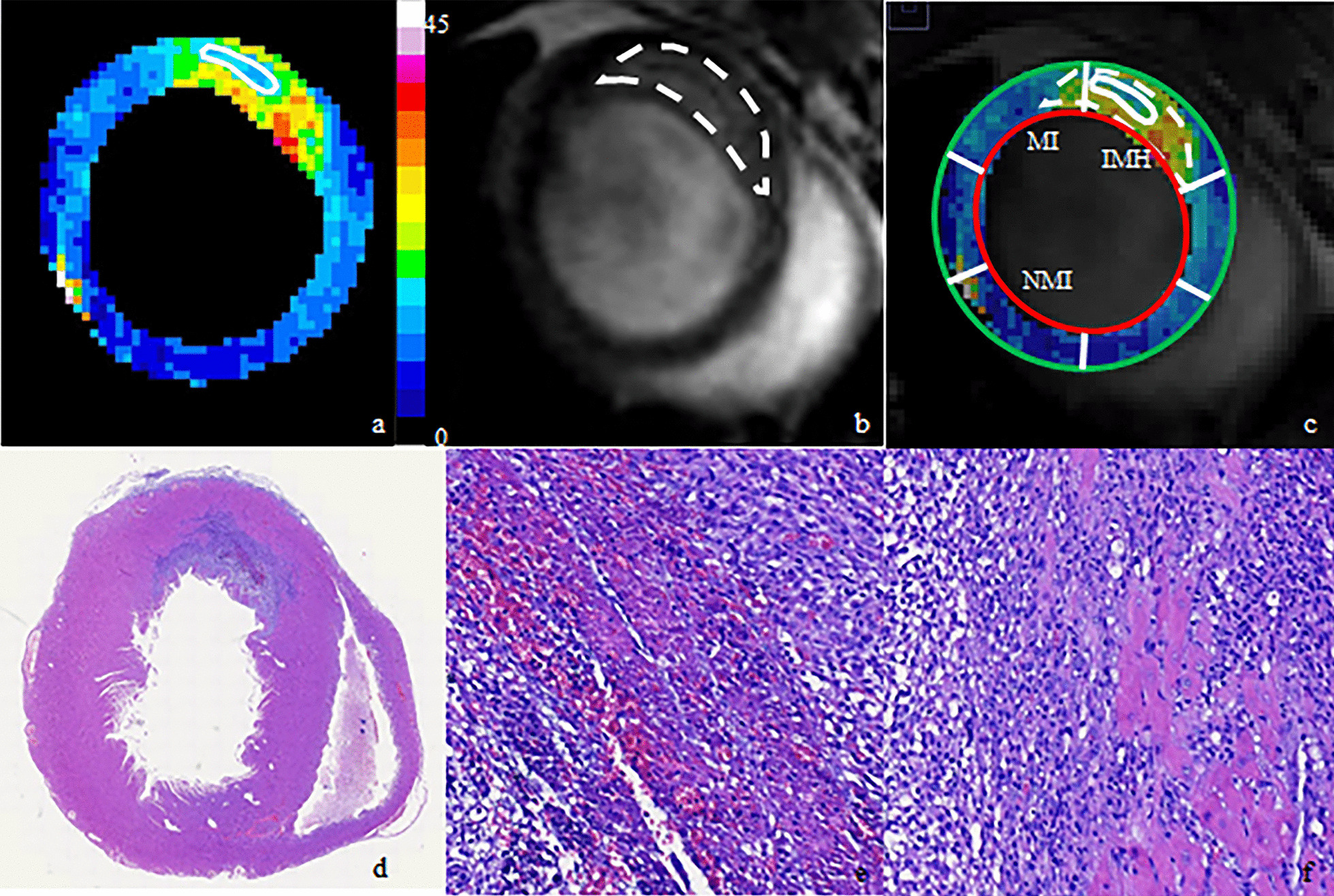
IMH, MI and NMI areas showed by MRI and histological examination. **a** T2* mapping demonstrated the IMH area (white enclosed area). **b** LGE sequence showed the myocardial infarction area (white dotted enclosed area). **c** The overlay of a and b showed the IMH, MI and NMI segments which was used for strain analysis. **d** The examination of H&E staining confirmed the incidence of IMH (**e**, × 20) and MI (**f**, × 20). *IMH* intramyocardial hemorrhage, *MI* myocardial infarction, *NMI* no myocardial infarction, *H&E* hematoxylin and eosin

T2* maps were calculated using custom-made software written in Matlab 7.1 (The MathWorks, Inc.). More than five slices (slices with poor image quality due to motion artifacts were excluded from analysis) were selected to decide the regions of hemorrhage for each rat, which identified as a hypointense core within a hyper intense territory on T2* maps. In the sham group, the myocardium was segmented into six segments (Figs. 2, 3).

The hyperenhanced myocardium on LGE images was defined as an MI area; this was detected using a computer-aided threshold of > 5 standard deviations (SDs) from the remote myocardium and adjusted manually and quantified using the multiple short-axis slice view. NMI areas were defined as segments without IMH and MI (The segments close to MI and IMH were exclusion for depressed strain compared with remote myocardium [11]. The MI and IMH distribution (segments) were evaluated by two experienced observers who were blinded to the experiment design. Discrepancies between the two observers were referred to another trained radiologist combined with the histopathologic results.

Normality was assessed using Kolmogorov–Smirnoff tests. Normally distributed data were expressed as mean ± SD, and comparisons between the groups were conducted using analysis of variance. Nonparametric data were expressed as median (25%–75% interquartile range) and compared using the Kruskal–Wallis test. *P* values of < 0.05 were considered statistically significant.

## Results

### Animals

MRI evaluations were successfully completed in 18 rats (9 in the reperfusion group and 9 in the sham group. Data from one rat was excluded because of death during surgery. Moreover, strain analysis was performed on 258 of 270 (95.6%) of the segments in the sham group and 158 of 270 (58.5%) of the segments of the reperfusion group (IMH = 19; MI = 79; and NMI = 60). Table 1 shows the changes of the heart rate of the sham and reperfusion groups.

**Table 1:** The changes of heart rate of the sham group and reperfusion group

	pre-CMR/bpm	pre-LDD/bpm	2 min post-LDD/bpm	20 min post-LDD/bpm	30 min post-LDD/bpm	*P*
Sham group	334 ± 20	261 ± 13	350 ± 29	324 ± 14	287 ± 12	< 0.01
Reperfusion group	363 ± 19	269 ± 31	351 ± 14	332 ± 23	299 ± 20	< 0.01

*CMR* cardiovascular magnetic resonance, *LDD* low-dose dobutamine

### The sham group

The PGRS and PGCS increased after LDD injection in the sham group (Table 2). In addition, the segmental PRS and PCS increased after dobutamine injection, and the EPRS and EPCS increased (Table 3).

**Table 2:** The global strain parameters of the sham group and the reperfusion group

	PGRS/%			PGCS/%		
	Rest	Stress	*P*	Rest	Stress	*P*
The sham group	36.8 ± 7.1	46.8 ± 8.6	< 0.05	− 19.6 ± 2.4	− 22.0 ± 2.7	< 0.05
The reperfusion group	35.7 ± 8.1	34.5 ± 12.5	0.734	− 18.8 ± 3.1	− 17.6 ± 5.1	0.337

*PGRS* peak global radial strains, *PGCS* peak global circumferential strains

**Table 3:** The strain parameters of the sham group

	PRS/%(M(P_25_,P_75_))			PCS/%(M(P_25_,P_75_))		
	Rest	Stress	*P*	Rest	Stress	*P*
Strain	36.1 (24.6,50.6)	50.4 (33.4,64)	< 0.01	− 20.2 (− 15.8, − 23.9)	− 24 (− 19.3, − 26.5)	< 0.01
The efficiency of strain	157.9 (107,219.9)	273.3 (183.2,344.1)	< 0.01	− 87.8 (− 68.2, − 104.4)	− 130 (− 104.1, − 143.2)	< 0.01

*PRS* peak radial strains, *PCS* peak circumferential strains

### The reperfusion group

The PGRS and PGCS did not increase after LDD injection in the reperfusion group (Table 2). The PRS and PCS of the IMH segment did not increase after dobutamine injection, and the EPRS and EPCS did not change (Table 4). Moreover, the PRS and PCS of the MI and NMI segments did not increase after dobutamine injection; however, the EPRS and EPCS increased (Table 5).

**Table 4:** Myocardial radial strains and circumferential strains in different segments of reperfusion group

Segment	PRS/%(M(P_25_,P_75_))			PCS/%(M(P_25_,P_75_))		
	Rest	Stress	*P*	Rest	Stress	*P*
IMH	30.1 (21.2,36.9)	25.2 (12.7,34.3)	0.295	− 16.8 (− 14, − 19.1)	− 14.6 (− 9.7, − 18.7)	0.198
MI	35.2 (24.5,50.9)	27.9 (21,57.8)	0.387	− 19.4 (− 15, − 24.2)	− 17.1 (− 13.9, − 24.9)	0.171
NMI	39.2 (21.7,64.1)	35.8 (21.4,69.1)	0.26	− 21.4 (− 13.4, − 26.8)	− 18.7 (− 13.1, − 26.6)	0.317

*PRS* peak radial strains, *PCS* peak circumferential strains, *IMH* intramyocardial hemorrhage, *MI* myocardial infarction, *NMI* non-myocardial infarction

**Table 5:** The efficiency of radial strains and circumferential strains in different segments of reperfusion group

Segment	EPRS/%/s(M(P_25_,P_75_))			EPCS/%/s (M(P_25_,P_75_))		
	Rest	Stress	*P*	Rest	Stress	*P*
IMH	128.5 (90.3,157.9)	132.3 (75.2,184.8)	0.841	− 77.2 (− 59.6, − 84.8)	− 81.8 (− 56.9, − 100.4)	0.334
MI	161.4 (104.2,230.7)	160.4 (116.2,302.3)	< 0.05	− 94.5 (− 65.9, − 117.2)	− 96.2 (− 77.2, − 135.5)	< 0.05
NMI	168.4 (92.7,283.7)	191.4 (117.8,371.1)	< 0.05	− 90.9 (− 61.8, − 117)	− 104.8 (− 71.5, − 144.6)	< 0.01

*EPRS* efficiency of peak radial strains, *EPCS* efficiency of peak circumferential strains, *IMH* intramyocardial hemorrhage, *MI* myocardial infarction, *NMI* non-myocardial infarction

The strains and efficiency of strain of the IMH segment were lower than those of the MI and NMI segments both before and after dobutamine injection (all *p* < 0.05). No significant difference was observed between the strains and efficiency of strain of the MI and NMI segments (all *p* > 0.05).

### The differences between the two groups

The strains and efficiency of strain of the IMH segment were lower than those of the sham group both before and after dobutamine injection (all *p* < 0.05). Additionally, the PCS and EPCS of the MI segment were lower than those of the sham group both before and after dobutamine injection (all *p* < 0.05). No significant differences in the PRS and EPRS were found between the MI segment and the sham group before dobutamine injection (both *p* > 0.05), but differences were found after LDD injection (both *p* < 0.05). No significant differences in the strains and efficiency of strain were observed between the NMI segment and the sham group before dobutamine injection (both *p* > 0.05); however, differences were found after LDD injection (both *p* < 0.05).

### Histopathology

Representative histopathologic images from animals with hemorrhagic infarctions sacrificed are shown in Fig. 3. H&E stains easily showed evidence of myocardial injury (myocyte necrosis), the distribution of inflammatory cells and hemorrhage.

## Discussion

This is one of a few studies using the CMR-FT technique in small animal models. In particular, we applied this technique to evaluate the segmental strain dysfunction in myocardium with IMH and MI in a rat model of acute reperfusion injury by using the LDD stress, we found that LDD would increase the PGRS and PGCS in normal sham rats, but not those in rats with acute reperfusion MI. And EPRS, EPCS of the MI/NMI segments increased, whereas those of the IMH segment remained the same. Interestingly, no difference in the PRS and PCS was found between the MI and NMI segments.

Dobutamine significantly increased the heart rate and the maximum circumferential strain even at low doses [12], which improve cellular energetics and contractile function in the hypoperfused myocardium. LDD was thus used to discriminate between viable and nonviable myocardia and the segmental and global functional recovery of patients with ischemic cardiomyopathy [13]. LDD stress CMR has good specificity (83%) and moderate sensitivity (74%) for detecting myocardial ischemia, similar to those of stress dobutamine echocardiography [14].

In this study, during anesthesia, the heart rate decreased from 310–380 to 250–270 bpm, and dobutamine was helpful in recovering the heart rate to normal ranges; therefore, promoting the efficiency of myocardial strain except for the strain value in normal rats. Furthermore, detecting the MI and NMI segments of the reperfusion group with the efficiency of myocardial strain became more sensitive than the strain value after dobutamine injection.

Limited data exist on the relationship between IMH and regional function. Kidambi et al. studied the role of myocardial deformation using tissue tagging-derived strains in patients with acute reperfused MI [4]. They demonstrated that regional function is poor in myocardial segments with IMH. In an echo study, Zhao et al. found that the segments of IMH had significantly decreased circumferential strains, but not longitudinal and radial strains [15]. Our study found that the PCS and EPCS of the MI segment were lower than those of the sham group without dobutamine stress, but not the PRS and EPRS. While the underlying mechanism is unknown, circumferential strain appeared to be more sensitive in assessing the infarct extension by identifying subtle impairments in LV contractile function. In this study, both peak strain values reduced in the IMH segments, compared to those values in MI and NMI segments. During the LDD stress, efficiencies of both strains in MI and NMI segments increased significantly, but no differences in these efficiencies were shown in the IMH segments. This finding indicates that efficiency of strain, with the use of LDD stress, could be a useful imaging biomarker in a clinical setting for identifying salvaged myocardial recovery after reperfusion, especially in the presence of IMH. Nevertheless, longitudinal studies in the future will be needed to test this hypothesis..

Interestingly, we found no difference in the PRS and PCS between the MI and NMI segments during LDD stress. Two possible explanations exist for this result: First, the deterioration of the strains in the MI segments may affect not only the segment itself but also neighboring segments. Second, strain parameters describe myocardial deformation, which is also determined by passive movement.

Our study has some limitations. First, this study had a small sample size; however, a study demonstrated that myocardial strain parameters were highly reproducible even with a small number of animals [8]. Therefore, no subgroups were set up for the MI segments according to the MI size. A larger sample size may be required to detect more subtle differences. Second, this study reported IMH only, not MVO; the reason was that FISP-cine with a relatively long acquisition time in the present study may underestimate the extent of MVO. Furthermore, the infusion method of LDD using veins was not performed in rats with small blood volumes [16]. Finally, we did not use cine in the long axis to calculate the longitudinal strain.

## Conclusions

In conclusion, this study demonstrated a method in which LDD could be useful in assessing the stain dysfunctions in segments with IMH, especially using the parameter of the efficiency of strain. IMH could be a crucial factor that decreased segmental contractility to a greater extent, thus further reducing the global function. A combination of LDD-CMR and LGE-CMR may be a powerful tool for identifying which segment with impaired LV function will benefit from revascularization.

## Data Availability

All data generated or analysed during this study are included in this published article.

## Abbreviations

IMH: Intramyocardial hemorrhage
MI: Myocardial infarction
LV: Left ventricular
LDD: Low-dose dobutamine
FT: Feature-tracking
NMI: Non-myocardial infarction
LGE: Late gadolinium enhancement
FISP: Fast imaging with steady-state precession
PGRS: Peak global radial strains
PGCS: Peak global circumferential strains
PCS: Peak circumferential strains
PRS: Peak radial strains
SDs: Standard deviations
MVO: Microvascular obstruction
EPRS: The efficiency of peak radial strains
EPCS: The efficiency of peak circumferential strains
